# Professional Care Team Burden (PCTB) scale – reliability, validity and factor analysis

**DOI:** 10.1186/s12955-014-0199-8

**Published:** 2015-02-07

**Authors:** Stefanie Auer, Elmar Graessel, Carmen Viereckl, Ursula Kienberger, Edith Span, Katharina Luttenberger

**Affiliations:** Department for Clinical Neurosciences and Preventive Medicine, Danube University Krems, Dr.-Karl-Dorrek-Straße 30, A-3500 Krems, Austria; Centre for Health Services Research in Medicine, Department of Psychiatry and Psychotherapy, Friedrich-Alexander-Universitaet Erlangen-Nuernberg, Schwabachanlage 6, 91054 Erlangen, Germany; MAS Alzheimerhilfe, Lindaustraße 28, A-4820 Bad Ischl, Austria

**Keywords:** Formal caregivers, Burden scale, Dementia

## Abstract

**Background:**

There is growing concern about how to provide care for persons with dementia in institutions such as nursing homes, day care centers, mobile services and hospitals. Care teams (formal caregivers) have to meet specific expectations from different sides: the Person with Dementia herself, the institution, and *from* different family members. Out of this situation, considerable burden can emerge hindering the professional development of care team members and counteracting quality of care of care recipients. So far there are very few specific reliable and valid scales measuring burden in care team members. Based on the theoretical concept of subjectively perceived burden, organizationally based factors of burden and structural factors of burden, we report on the construction of a care team burden scale and its scale quality criteria.

**Methods:**

*Based on the theoretical three assumed sources of burden*, a structured interview guide was developed. Interviews were held with professional caregivers. Through qualitative data analysis, an item pool consisting of 40 Items was constructed. Experts selected 19 items found most appropriate to measure the three theoretically based domains of burden. The Perceived Stress Scale (PSS) was chosen as a criterion in order to test *discriminant* validity. An exploratory factor analysis was performed.

**Results:**

The stepwise scale analysis revealed a 10 item solution. The Cronbach’s alpha was *0.785*. The Pearson correlation between the PCTB 10 Item scale (mean score 10.2, SD = 5.0) and the PSS (mean score 13.0, SD = 5.9) was 0.46 (p < 0.001). All included items could clearly be assigned to one of three factors.

**Conclusion:**

The 10 item PCTB scale provides a valid and reliable means of obtaining ratings *of burden* from formal care teams working in *nursing homes* in order to evaluate different interventions targeted at the reduction of burden in care teams.

**Electronic supplementary material:**

The online version of this article (doi:10.1186/s12955-014-0199-8) contains supplementary material, which is available to authorized users.

## Background

The WHO predicts 115 million Persons with Dementia in 2050 [1]. A significant fraction of affected persons will be cared for in different institutional settings and care provision services such as nursing homes, day care centers and mobile care services. Care teams working in these settings are put under considerable pressure [2], resulting in a burdened and stressed work force. Work related stress and burden results in high turnover [3-5], low morale and increased sick leave [6], seriously acting against quality care and resulting in staff shortage. Caring for care teams seems an important issue in the face of intensive need of institutional care in the future [7,8]. More generally, caregiver burden is defined as “Alterations in caregivers’ emotional and physical health, which can occur when care demands outweigh available resources” [9]. Traditionally, burden has been especially investigated in family caregivers [10-12] with the goal of providing *family caregivers* with appropriate support and to *developing* appropriate counselling programs [13]. *It has been suggested that caregiver burden be assessed routinely* [14]. Considering today’s high expectations put on institutional care with respect to “person centred care” [15] and the related skills of understanding the needs of persons with dementia beyond regular *physical* care, a high emotional investment on the side of care teams is required. This may make the two caregiving experiences comparable even though essential differences exist [16].

Research starts to uncover the relationships between staff behavior and the behavior of care recipient [17,18]. Training and coaching programs have an effect on caregiver stress [19], however, the exact content and didactic procedures need to be developed and the effect on care team burden and quality of life for care recipients studied. Studies investigating subjective burden in informal caregivers *found* stress related symptoms like higher caregivers’ emotional and physical complaints [20], higher incidence of behavioral problems and falls of the care-*recipient* [18], and higher risk for abusive behavior in the case of caregivers with higher burden scores [21]. These results give way to many treatment ideas and interventions for institutional settings. There is a lack of brief and practical scales measuring different aspects of burden in professional care teams. *There are very few scales specifically addressing caregiver burden in professional teams of nursing homes. Existing scales tend to concentrate on one aspect of burden- for example behavioral problems* [22]*.* It was our intention to develop an instrument with a broader burden concept. In the literature, there are three main sources of burden identifiable. Firstly, subjective sources of burden for which internal individual factors such as personality structure, life experience, motivation, attitude towards Persons with Dementia, education and life situation are made responsible [23]. Secondly, objective factors of burden that are related to disease symptoms (e.g. problem behaviours and the decline of functions). Thirdly, structural sources of burden related to work conditions such as lifting heavy persons, architectural problems, time problems, *organizational culture* [24]. On the basis of these three theoretically defined sources of burden, the Professional Care Team Burden (PCTB) scale was developed. This investigation describes the psychometric properties, item performance, first reliability and validity information and the factor structure of the scale.

## Methods

### Scale construction

The steps of scale development are depicted in Table 1.

**Table 1:** **Steps of scale construction**

Step 1	Selecting the theoretical basis
Step 2	Performing interviews with professional caregivers
Step 3	Analysis of interviews and constructing an item pool of 40 item suggestions
Step 4	Experts select appropriate items (19 items research version)
Step 5	Stepwise item reduction
Step 6	10 item scale

With the underlying theoretical model of burden, a structured interview containing seven questions and covering the three sources of burden was developed. *In order to achieve a representative sample of care staff, two institutions (one privately and one state owned) employing 60 care persons of different educational levels (nurses aid, nurse) were approached and asked whether 10 persons could be selected by chance for an interview on staff burden. The care persons selected were all female (4 registered nurses, 6 nurse’s aides; age 28–55 years).* The interviews were recorded and transcribed. From the transcriptions, 40 potential topics were generated using the Mayring method *of qualitative content analysis* [25]. From these topics, preliminary items were formulated. Three experts (*a Geriatrician, a Clinical Psychologist and a Social Worker*) independently selected an equal amount of appropriate items for the assessment of the three dimensions of burden. The selections then were reviewed together and a consensus about the items selected was reached. The first raw scale version consisted of 19 items (7 items from the construct perceived subjective burden, 6 items from the construct perceived objective burden and 6 items from the construct perceived structural burden). An additional open question (item 20 “Are there any other areas you find burdening, that have not been mentioned? If yes, which areas?”) was added *to the research scale version*. The list of items as it was used in the study is depicted in Table 2.

**Table 2:** **Research version of the PCTB used in the first reliability and validity study**

**No.**	**Item** ^**+**^
1	In caring for residents I am able to adequately respect the needs of the person
2	In my daily routine I find time to recover
3	My work performance is respected by my colleagues
4	Because of my education and my professional routine I am able to solve my work challenges
5	In my daily routine I am sometimes insecure about the areas of my competency*
6	I can discuss work related issues with my colleagues
7	I feel that the contact with my superiors is good
8	I can participate in organizing the daily routine in my organization
9	I can handle the physical aspects of care (carrying, lifting, hot bathing areas)
10	The loss of ability to communicate in persons with dementia bothers me*
11	I can manage behaviours resulting from disorientation in persons with dementia
12	Difficult behaviours (aggression, wandering) of persons with dementia are difficult to bear*
13	I can accept and bear illness and death of older people in the circle of life
14	To observe how persons with dementia are getting worse makes me sad*
15	I am able to contribute to a positive working climate
16	In my daily work I sometimes feel *worn out* and depressed*
17	I can handle constructive critique
18	I can keep personal problems out of my daily work routine
19	My personal life/family environment is supportive and is able to unburden me
20	Are there any other areas that you find burdening that have not beet qualitative mentioned? If yes, which areas?**

^+^Items are based on a structured Interview using the following questions: 1. How do you do at work in terms of time management? 2. What are the expectations towards you posed from your superiors of the organization? 3. How do you feel about the working climate in your institution? 4. How do you feel about the daily suffering of the persons with dementia and their relatives under your care? 5. How do you feel about the possibility to recover on your work-free days? 6. What do you appreciate in your work? What are the parts of your work that you do not appreciate? 7. Are there any other factors burdening you in your daily routine that you would like to mention?

*Negatively poled items.

**Qualitative question.

A five-point response scale (strongly agree, agree, neutral, disagree, and strongly disagree) was defined *(scores ranging from 0 to 4).* The maximum score of this version was 76. In order to reduce the possibility of a *response bias*, 5 items were negatively poled (Item 5, 10, 12, 14 and 16). The research scale was constructed in German. The German items were translated into English and back-translated into German (the German version is available in Additional file 1). For the validity and reliability study, basic socio demographic features (gender, age, years of employment and educational level) were assessed. As a criterion for the *discriminant* validity assessment, the 10 item version of the PSS (Perceived Stress scale) [26] was used. *“The PSS is an index of general stress appraisal and measures the degree to which situations are perceived as stressful”. Scores can range from 0 to 40, with higher scores indicating greater stress”.* T*o study the scale quality criteria, the newly developed scale and the PSS was presented to 13 different nursing homes (employing about 390 care persons) in different Austrian counties by a master student (SS) of the dementia studies at the Danube University in Austria. The student sent the questionnaires to her study colleagues working in different care institutions (privately and state owned institutions) asking for distribution. The questionnaire was introduced in team meetings and displayed in staff rooms. Participation was voluntary and anonymous. The filled out questionnaire was collected anonymously in the staff room and sent back to the master student.* An introductory text explained the study purpose and guaranteeing anonymity *to the person filling out the questionnaire*.

### Statistical analysis

A stepwise item selection procedure was performed based on the item quality, taking the results of the internal consistency analysis as a measure of reliability [27] into account. *Discriminant* validity was obtained using the Perceived Stress Scale (PSS) as a criterion and calculating the correlation coefficient. The significance level was alpha = 0.01. A threshold of >0.3 for *corrected item-total-correlation* was chosen sufficient. Items were eliminated if their elimination caused an increase in the Cronbach’s Alpha value. The theoretical basis of the scale was tested applying a principal component analysis with orthogonal rotation (VARIMAX). Communalities bigger than 0.5 were accepted since the sample was bigger than 100 persons [28]. The Kaiser-Meyer-Olkin criterion was used to test the requirements for a factor analysis [29]. *Missing values were substituted using the individual mean score of each total scale score.* Items not clearly loading on any factor were excluded. Statistical Analysis was performed using the SPSS Vol. 19.0 for Windows.

### Ethical considerations

Participants were informed about the intent of the study and the participation in the study was on a voluntary basis only. Confidentiality was guaranteed to participants. Questionnaires were recollected anonymized (without names) and participants could not be identified by their questionnaires. Consequently, data analysis was also performed in an anonymous fashion.

The method of questioning care personnel was approved by the ethics committee of Upper Austria (Study Nr.M-2-12, 18.4.2012).

## Results

### Study population

*172 persons filled out the questionnaire (response rate of 44.1%)*. 140 persons (81.4%) were female, 27 persons (15.7%) were male and 5 persons (2.9%) did not disclose their sex. The mean age of the population was 43.0 years (SD = 10.1; min = 19, max = 60), 11 persons did not disclose their age. 98 persons (57.0%) had a nursing degree, 71 (41.3%) were nurses assistants. 3 persons did not disclose their educational level. 13 persons (7.5%) were working less than two years in their institution, 55 (32.0%) were working between three and ten years in the institution and 95 persons (55.3%) were working more than ten years in their respective institutions. 9 persons (5.2%) did not answer this question.

### Reliability and validity of the PCTB research version

*The open qualitative research question was excluded from further analysis since this question was intended to serve as a “research item” only. 9 diverse categories emerged from the answers. The most frequently named areas were lack of time (10 persons), work schedule (2 persons), conflicts with colleagues or family members (2 persons), missing respect by superiors (2 persons), young onset dementia (1 person), lack of practical experience of superiors (2 persons), dementia in the family (1 person), missing supervision for personnel (1 person), time consuming documentation (1 person)*. The results of the remaining 19 items revealed a Cronbach’s alpha of 0.834. The Pearson correlation between the 19 items research version (mean score = 10.2, SD = 5.0) and the PSS (mean score = 13.0, SD = 6.0) as a measure of *discriminant* validity was 0.35 (p < 0.001). Items showing little *corrected item-total-correlation* were eliminated, also items, causing an increase in Cronbach’s alpha if excluded (Items Nr. 5, 13 and 14). The elimination of these three items caused an increase of the Cronbach’s alpha to 0.846.

### Factor analysis of the PCTB research version

The principal component analysis with VARIMAX rotation was performed for the remaining 16 items *(6 items from the construct subjective burden, 4 items from objective burden, 6 items from structural burden)*, resulting in a four factor solution: Factor 1 (structural burden; Items 3, 6, 7, 8; eigenvalue = 5.109), Factor 2 (objective burden; Items 10, 11, 12, 16; eigenvalue = 1.276), Factor 3 (subjective burden; Items 17, 18, 19; eigenvalue = 1.015). Factor 4 (Items 1, 2, 4, 9; eigenvalue = 1.615) could not be interpreted since there was no common theme found. In addition, the responses of these items tended to produce “no burden” responses. Therefore these items were excluded. Item 15 (“I am able to contribute to a positive working climate”) did not load on any factor and was eliminated as well. Item 16 (“In my daily work I sometimes feel *worn out* and depressed”) loading on factor 3 was eliminated because it did not fit the rest of the factor theme. As a result, the final version of the scale consisted of 10 Items. We performed a second analysis of scale criteria with the 10 Item scale version. The mean scores depicted in Table 3 reveal, that the *whole range of response options* was used in this population. *The pre-defined categorization of three out of six (50%) items from the dimension subjective burden, three out of four (75%) items from objective burden and four out of six (66.66%) items from structural burden could be confirmed by the factor analysis*.

**Table 3:** **Subscale and item characteristics of the 10 items PCTB scale (** ***N*** **= 172)**

		***n*** **(%)**						***Corrected r(it)***	**Cronbach’s alpha, if item is deleted**
**Subscales and items**		***Strongly disagree***	***Disagree***	***Neutral***	***Agree***	***Strongly agree***	***Missing***		
*Structural Burden (Cronbach’s Alpha = .784)*									
	1. My work performance is respected by my colleagues.	0 (0%)	6 (3.5%)	42 (24.4%)	81 (47.1%)	39 (22.7%)	4 (2.3%)	.541	.756
	2. I can discuss work related issues with my colleagues.	1 (0.6%)	6 (3.5%)	17 (9.9%)	76 (44.2%)	70 (40.7%)	2 (1.2%)	.582	.737
	3. I feel that the contact with my superiors is good.	3 (1.7%)	5 (2.9%)	21 (12.2%)	72 (41.9%)	71 (41.3%)	0 (0%)	.592	.731
	4. I can participate in organizing the daily routine in my organization.	4 (2.3%)	15 (8.7%)	28 (16.3%)	70 (40.7%)	51 (29.7%)	4 (2.3%)	.664	.694
*Objective Burden (Cronbach’s Alpha = .711)*									
	5. The loss of ability to communicate in persons with dementia bothers me.*	26 (15.1%)	70 (40.7%)	47 (27.3%)	22 (12.8%)	6 (3.5%)	1 (0.6%)	.571	.568
	6. I can manage behaviours resulting from disorientation in persons with dementia.	0 (0%)	5 (2.9%)	31 (18%)	98 (57%)	35 (20.3%)	3 (1.7%)	.513	.666
	7. Difficult behaviours (Aggression, Wandering) of persons with dementia are difficult to bear.*	30 (17.4%)	71 (41.3%)	43 (25%)	23 (13.4%)	5 (2.9%)	0 (0%)	.544	.609
*Subjective Burden (Cronbach’s Alpha = .550)*									
	8. I can handle constructive critique.	0 (0%)	2 (1.2%)	25 (14.5%)	101 (58.7%)	43 (25%)	1 (0.6%)	.364	.456
	9. I can keep personal problems out of my daily work routine.	2 (1.2%)	3 (1.7%)	11 (6.4%)	90 (52.3%)	66 (38.4%)	0 (0%)	.357	.455
	10. My personal life/family environment is supportive and is able to unburden me.	2 (1.2%)	7 (4.1%)	22 (12.8%)	59 (34.3%)	82 (47.7%)	0 (0%)	.381	.431

*Note. Corrected r(it* **)** = corrected item-total-correlation.

*Negatively poled items (Item Nr. 5 and 7): strongly disagree = 0, disagree = 1, neutral = 2, agree = 3, strongly agree = 4.

All other items are positively poled: strongly disagree = 4, disagree = 3, neutral = 2, agree = 1, strongly agree = 0.

### Reliability and validity of the 10 item PCTB

The Cronbach’s alpha of the 10 item PCTB scale was recalculated and revealed a value of *0.785. The Cronbach’s alpha was calculated for the three subscales and revealed values of 0.784 for Structural Burden, 0.711 for Objective Burden and 0. 550 for Subjective Burden.* The Pearson correlation between the 10 Item PCTB scale (mean score 10.2, SD = 5.0) and the PSS (mean score 13.0, SD = 5.9) was 0.46 (p < 0.001). *For the subscales*, *Structural Burden (mean score 3.8, SD = 2.7), Objective Burden (mean score 3.9, SD = 2.2) and Subjective Burden (mean score 2.4, SD = 1.7), the Pearson correlation with the PSS was 0.27 (p = 0.001), 0.44 (p < 0.001) and 0.36 (p < 0.001) respectively. The correlation for Structural Burden (0.27) and Objective Burden (0.44) were significantly different (Z = 1.8, p < 0.05). The correlation coefficients of Subjective Burden and Objective Burden however were not significantly different.* All Items were in the >0.3 *corrected item-total-correlation* range.

### Factor analysis of the 10 item PCTB

For the factor analysis, all requirements were fulfilled. The Kaiser-Meyer-Olkin criterion was fair (0.762). The Bartlett test was significant (Chi^2^_df=45_ = 430.21; p < 0.001). According to the Kaiser-Criterion three factors were extracted. On the VARIMAX rotation method, all items showed clear loadings (>0.60) on one of the three factors (see Table 4).

**Table 4:** Results of the VARIMAX rotated factor loading matrix for the PCTB**

**Item**	**Factor**		
	**1**	**2**	**3**
	**“Structural”**	**“Objective”**	**“Subjective”**
(3) My work performance is respected by my colleagues.	**0.70**	0.16	0.20
(6) I can discuss work related issues with my colleagues.	**0.80**	0.07	0.00
(7) The feel that the contact with my superiors is good.	**0.73**	0.10	0.22
(8) I can participate in organizing the daily routine in my organization.	**0.81**	0.18	0.07
(10) The loss of ability to communicate in persons with dementia bothers me.*	0.10	**0.78**	0.17
(11) I can manage behaviours resulting from disorientation in persons with dementia.	0.20	**0.71**	0.22
(12) Difficult behaviours (Aggression, Wandering) of persons with dementia are difficult to bear.*	0.14	**0.82**	−0.04
(17) I can handle constructive critique.	0.16	0.07	**0.69**
(18) I can keep personal problems out of my daily work routine.	0.05	0.39	**0.60**
(19) My personal life/family environment is supportive and is able to unburden me.	0.12	0.02	**0.79**

*Negatively poled items, **highest loadings are printed in bold face.

On the factor “structural burden” (eigenvalue = 3.434), Items 3, 6, 7 und 8 loaded, on the factor “objective burden” (eigenvalue = 1.468), the items 10, 11 und 12 loaded, and on the factor “subjective burden” (eigenvalue = 1.157), the items 17, 18 and 19 loaded. The final version of the scale is presented in Table 5 (German version see Additional file 1). The total maximum burden score of the 10 Item PTB scale is 40.

**Table 5:** **Professional care team burden scale (PCTB) – 10 item version**

	**Description**	**Strongly disagree**	**Disagree**	**Neutral**	**Agree**	**Strongly agree**
Item 1:	My work performance is respected by my colleagues.	□	□	□	□	□
Item 2:	I can discuss work related issues with my colleagues.	□	□	□	□	□
Item 3:	The contact with my superiors is good.	□	□	□	□	□
Item 4:	I can participate in organizing the daily routine in my organization.	□	□	□	□	□
Item 5:	The loss of ability to communicate in persons with dementia bothers me *.	□	□	□	□	□
Item 6:	I can manage behaviours resulting from disorientation in persons with dementia.	□	□	□	□	□
Item 7:	Difficult behaviours (Aggression, Wandering) of persons with dementia are difficult to bear.*	□	□	□	□	□
Item 8:	I can handle constructive critique.	□	□	□	□	□
Item 9:	I can keep personal problems out of my daily work routine.	□	□	□	□	□
Item 10:	My personal life/family environment is supportive and is able to unburden me.	□	□	□	□	□

*Negatively poled items.

All the raw data of this analysis are made available in Additional file 1.

## Discussion

In this study, a 19 items research scale version (plus one additional qualitative item) constructed from an item pool was stepwise reduced to a 10 item burden scale named PCTB scale. *The qualitative item was intended for the research scale version in order to explore further important themes. It was not added to the final scale version*. Even though the results of the 10 item version need to be considered preliminary, since both steps of scale quality analysis were performed on the basis of the same data sample, they are promising. *Cronbach’s alpha for the entire scale as a measure of internal consistency was 0.785, indicating a high reliability. Cronbach’s alpha of the three underlying scale constructs were lower than the overall value supporting the original hypothesis of combining structural, objective and subjective sources of burden. However,* future studies have to investigate the test-retest reliability. The correlation coefficient between the 10 item PCTB and the PSS was 0.46. *This value represents a fair support for discriminant validity. However, the correlations of the subscales of the PCTB with the PSS were all lower* (0.27, 0.44 and 0.36) supporting a difference between the two concepts of stress and burden. *The concepts are interrelated as some items in both scales assess an individual’s sense of control and competence. The subscale “structural burden” correlates the lowest with the PSS and introduces new important themes of team culture. The PSS has been used to assess stress levels in care teams in an unspecific fashion* [19]*. This procedure however does not provide an insight into the specific sources of stress. The intention of the PCTB is to assess burden in care teams related to the care for persons with dementia in a specific manner. This could have clear advantages over non- specific scales as the PCTB may be able to uncover areas for intervention. Further, this specificity may be better accepted by care teams since the relevance to their daily routine is clearly visible. However, future research needs to confirm this*.

The hypothesis, that three sources are equally responsible for the subjectively perceived burden of professional care teams was supported by the three factor solution found in this study. With this result, the PCTB with its broad concept of burden could be used in different settings as a screening research and diagnostic tool contrasting existing scales with a still more specific concept (for example behavioral problems only). *The three dimensional concept of the scale enables the scale to be used in other care settings such as day care centers and can also be used with care teams providing care at home*. However, the scale has not been tested for this population. In the construction process of the scale, rather positive formulations were preferred in order to promote a positive self- image *within a care team*. However, in order to prevent a specific *response bias*, and to give enough room to admit symptoms of burden, some questions were negatively poled. Future research on the PCTB scale should take into account different care settings, such as day care centers and formal residential care. A re-validation of the 10 item version of the PCTB scale in the different care environments should be striven for.

During this study we noticed, how reluctant some study participants were to disclose private information (age, professional background, sex). Some were concerned that the results could be provided to the organization. This perceived fear *within care teams* seems to point to a serious problem that future research needs to address since it may significantly interfere with work satisfaction. Research could help in developing new concepts of team formation and finding methods to unburden this neglected work force [30]. Caring for care staff, empowering this profession is one of the major challenges in the future endeavor of improving institutional care. Support structures for care teams need to be developed in order to make this profession more attractive. As measuring the burden of care of family members is recommended as a routine [14], we suggest approaching this issue in a similar manner for professional care teams. Considering the importance of institutional care in the future, the effect of different interventions should also take the subjective feelings of burden of the formal care staff members into account.

## Conclusions

This study provides preliminary scale quality data on a short practical scale measuring burden in professionals working in nursing homes supporting persons with dementia. The results are promising.

## Additional file

Additional file 1:
**Professional Care Team Burden Scale (PCTB) – 10 item version (German).**

## Abbreviations

PCTB: Professional Care team burden scale
PSS: Perceived Stress Scale
M-NCAS: Modified Nursing Care Assessment Scale
